# Cannabis use disorder and five-year risk of oral cancer in a multicenter clinical cohort

**DOI:** 10.1016/j.pmedr.2025.103185

**Published:** 2025-07-21

**Authors:** Raphael E. Cuomo

**Affiliations:** University of California, San Diego, School of Medicine, San Diego, CA, USA

**Keywords:** cannabis use disorder, Incidence, Oral cancer, Risk factors

## Abstract

**Objective:**

Cannabis use disorder (CUD) is increasingly prevalent in the United States, yet long-term health consequences remain poorly defined. Oral cancer is plausible given shared carcinogens between cannabis and tobacco. This study assessed associations between CUD and five-year oral cancer risk in a large clinical cohort.

**Methods:**

This retrospective cohort study analyzed clinical records from the University of California Health Data Warehouse, covering six academic medical centers. Adults screened for drug use disorders between January 2012 and December 2019 were included if they had no prior oral cancer diagnosis. The index date was the date of first screening. Patients were followed for five years for oral cancer diagnoses (lip or tongue), thereby extending data collection to December 2024. CUD was defined by a new ICD-coded diagnosis during follow-up. Logistic regression and Cox proportional hazards models estimated odds ratios (ORs), hazard ratios (HRs), and 95 % confidence intervals (CIs), adjusting for age, sex, body mass index (BMI), and smoking status.

**Results:**

Among 45,129 eligible patients, 949 (2.1 %) developed CUD. Oral cancer incidence was 0.74 % in the CUD group and 0.23 % in non-CUD patients. CUD was associated with significantly increased risk of oral cancer (unadjusted OR 3.24; 95 % CI, 1.50–7.00). The association remained significant after adjustment (adjusted OR 3.25; 95 % CI, 1.47–7.17; adjusted HR 3.25; 95 % CI, 1.48–7.13).

**Conclusions:**

CUD was linked to a more than threefold increase in oral cancer risk over five years. These findings highlight the need to assess long-term oncologic risks of problematic cannabis use.

## 1 Introduction

Cannabis use is increasingly common in the United States, yet its long-term health risks, particularly carcinogenic outcomes, remain uncertain. Cannabis combustion produces polycyclic aromatic hydrocarbons and other carcinogens similar to those found in tobacco smoke, including benzo[*a*]pyrene and phenols (Moir et al., 2008). While the association between tobacco and oral cavity cancers is well established, the oncogenic potential of cannabis remains less clearly defined, despite similar modes of administration and shared toxicants.

Epidemiologic studies investigating cannabis and cancer have yielded mixed results. Some observational studies suggest an elevated risk for head and neck cancers, particularly among frequent users, while others report null associations (Zhang et al., 2015; Ghasemiesfe et al., 2019). These discrepancies may stem from methodological limitations, including reliance on self-reported cannabis use, short follow-up periods, and lack of control for confounding variables such as tobacco and alcohol consumption. Furthermore, few studies have focused specifically on oral cancers, a biologically plausible site given the direct exposure of oral mucosa to smoke during cannabis inhalation.

Cannabis use disorder (CUD) is a clinical diagnosis reflecting problematic use and is associated with high-frequency consumption patterns conferring elevated exposure to cannabis compounds (Ghasemiesfe et al., 2019). However, CUD does not capture the full population of frequent cannabis users, many of whom do not meet diagnostic criteria (Callaghan et al., 2020). However, CUD is more reliably documented in electronic health records than self-reported use, with prior studies showing that a diagnosis of CUD based upon diagnostic thresholds from the.

Diagnostic and Statistical Manual of Mental Disorders (5th Edition) is more likely than not to be observed among individuals using approximately 14 joints per week (Callaghan et al., 2020). While specific data on inhalation volume or depth are not available in clinical records, increased frequency of cannabis use may lead to greater cumulative exposure to combusted cannabis compounds interacting with the oral mucosa. In the absence of structured clinical data on dose and frequency, CUD can serve as a pragmatic, if conservative, proxy for identifying patients likely to have high exposure.

Understanding whether CUD confers elevated cancer risk is valuable for informing clinical practice, especially as cannabis legalization expands and perceptions of risk decline. Therefore, to examine the association between CUD and subsequent risk of oral cancer, we conducted a retrospective cohort study using electronic health record data from a large, multisite academic health system in California.

## 2 Methods

This retrospective cohort study utilized electronic health record (EHR) data from the University of California Health Data Warehouse, a centralized data repository encompassing clinical records from six academic medical centers across California. Patients were eligible for inclusion if they completed a documented drug use disorder screening encounter between January 1, 2012, and December 31, 2019, and had no prior diagnosis of oral cancer at baseline.

The index date was defined as the date of the earliest recorded screening. Each patient was followed for a period of five years from their index date to determine the development of new-onset oral cancer, defined as malignant neoplasms of the lip or tongue (International Classification of Diseases, version 10 [ICD-10] codes C00–C02). Therefore, this study leveraged clinical record data through the end of 2024. The exposure of interest was CUD, operationalized using ICD-9/10 codes corresponding to cannabis abuse or dependence. Patients were categorized into the CUD group if they received a new CUD diagnosis at any time within the five-year follow-up period.

Demographic and clinical covariates included age at index date (continuous), sex (female vs. male), body mass index (BMI, continuous), and smoking status (ever vs. never smoker), derived from structured EHR fields. Multivariable logistic regression was used to estimate adjusted odds ratios (ORs) for oral cancer incidence, while Cox proportional hazards regression evaluated the association between CUD and time to cancer diagnosis. All statistical tests were two-sided with α = 0.05. Analyses were conducted in R version 4.2.3 using complete case data. This study is exempt from human subject protection under Institutional Review Board protocol 1604619-1 of the University of California Health System.

## 3 Results

A total of 45,129 patients met eligibility criteria, of whom 949 (2.1 %) were newly diagnosed with CUD during the five-year follow-up period. The remaining 44,180 (97.9 %) did not receive a CUD diagnosis. The cohort had a mean age of 44.5 years (SD = 14.1), and 54 % were female. Oral cancer was diagnosed in 106 patients over the follow-up period. Among those without CUD, the incidence of oral cancer was 0.23 %, consistent with population-level estimates. In contrast, 0.74 % of patients with CUD developed oral cancer.

Unadjusted logistic regression revealed that CUD was associated with significantly higher odds of oral cancer (OR 3.24; 95 % CI, 1.50–7.00). After adjusting for age, sex, BMI, and smoking status, the association remained significant (adjusted OR = 3.25; 95 % CI, 1.47–7.17; Table 1). Patients with CUD in this model were more often male (50.7 % vs 40.6 %), younger (mean age 51 vs 60), leaner (mean BMI 23.2 vs 24.1 kg/m^2^), and more likely to smoke tobacco (24.9 % vs 5.9 %) compared to those without CUD. All covariates exhibited a statistically significant association with oral cancer development with the exception of tobacco smoking, likely due to limited power as smokers constituted only 6.3 % of the total sample. A subgroup analysis was conducted among only tobacco smokers; smokers with CUD had significantly higher rates of oral cancer than smokers without CUD (adjusted OR = 6.24, 95 % CI 1.81–21.54) while controlling for sex, age, and BMI.

**Table 1:** Association between cannabis use disorder and five-year incidence of oral cancer among adults with no prior oral cancer who completed a documented drug use disorder screening in the University of California Health Data Warehouse (six academic medical centers in California; 2012–2024).

Covariate	Odds Ratio (OR)	95 % CI (Lower)	95 % CI (Upper)
Cannabis Use Disorder	3.25	1.47	7.17
Smoking	1.48	0.91	2.41
Age (years)	1.04	1.02	1.06
Female	0.97	0.95	0.99
BMI (kg/m^2^)	0.94	0.89	0.99

**Footnote:** Oral cancer includes malignant neoplasms of lip or tongue. Smoking indicates any documented history of tobacco smoking (current or former) in the electronic health record, with referent as no documented smoking. Results are from multivariable logistic regression adjusted for age, sex, body mass index, and tobacco smoking status. CUD = cannabis use disorder. BMI = body mass index (kg/m^2^). *p* values are two sided (Wald tests).

Similarly, in Cox proportional hazards modeling, the unadjusted hazard ratio (HR) for time to oral cancer diagnosis in the CUD group was 3.24 (95 % CI, 1.50–6.96). The adjusted hazard ratio remained statistically significant (HR 3.25; 95 % CI, 1.48–7.13). Age was also positively associated with cancer risk (adjusted HR per year increase = 1.04; *p* < 0.01), while higher BMI and female sex were associated with slightly lower risk (Table 2).

**Table 2:** Association between cannabis use disorder and time to incident oral cancer diagnosis within five years among adults with no prior oral cancer who completed a documented drug use disorder screening in the University of California Health Data Warehouse (six academic medical centers in California; 2012–2024).

Covariate	Hazard Ratio (HR)	95 % CI (Lower)	95 % CI (Upper)
Cannabis Use Disorder	3.25	1.48	7.13
Smoking	1.60	0.88	2.90
Age (years)	1.04	1.03	1.04
Female	0.97	0.96	0.99
BMI (kg/m^2^)	0.94	0.91	0.98

**Footnote:** Oral cancer includes malignant neoplasms of lip or tongue. Smoking indicates any documented history of tobacco smoking (current or former) in the electronic health record, with referent as no documented smoking. Results are from multivariable Cox proportional hazards regression adjusted for age, sex, body mass index, and tobacco smoking status. CUD = cannabis use disorder. BMI = body mass index (kg/m^2^). p values are two sided (Wald tests).

## 4 Discussion

In this large clinical cohort, patients who developed CUD after drug use screening had more than three times the odds of developing oral cancer within five years compared to patients who remained CUD-free. This association persisted after adjusting for age, sex, smoking status, and body mass index, supporting a robust relationship between CUD and subsequent oral cancer risk. Although the absolute incidence of oral cancer remained low, the relative risk increase was notable and consistent with existing toxicological concerns.

Cannabis smoke contains many of the same carcinogenic compounds found in tobacco smoke, including polycyclic aromatic hydrocarbons and volatile organic compounds, which have known mutagenic effects on epithelial tissue (Moir et al., 2008). Cannabis smoke has been shown to produce cytologic and histopathologic changes in respiratory tract cells similar to those caused by tobacco (Barsky et al., 1998). For example, bronchial biopsy studies of habitual marijuana users demonstrate epithelial dysplasia and metaplasia comparable to tobacco smokers, even in the absence of tobacco use (Barsky et al., 1998).

Animal and in vitro studies further support a carcinogenic potential for cannabis smoke. Cannabis condensates have been found to induce DNA damage and chromosomal aberrations in mammalian cells, as well as mutations in bacterial assays (Roth et al., 1998). In rodents, long-term inhalation of cannabis smoke has been associated with pre-malignant and malignant changes in pulmonary tissue (Roth et al., 1998).

The epidemiological literature has been mixed, partly due to varying definitions of exposure and limited sample sizes. Some studies have found an elevated risk of head and neck cancers among heavy or frequent cannabis users (Zhang et al., 2015; Berthiller et al., 2008); while others, particularly those unable to adjust adequately for tobacco or alcohol, have found no significant association (Ghasemiesfe et al., 2019). Our study differs in that it uses a defined clinical diagnosis (CUD), likely representing more sustained and problematic cannabis use than occasional or recreational consumption. This distinction may help explain the stronger association we observed.

Another possible mechanism is cannabis-induced immune suppression. Δ9-tetrahydrocannabinol, the primary psychoactive compound in cannabis, has been shown to suppress both innate and adaptive immune responses, including inhibition of natural killer cell activity and impaired cytokine signaling (Klein, 2005). Such effects may compromise immune surveillance and facilitate tumor initiation or progression, particularly in mucosal tissues directly exposed to smoke.

These findings may also have implications for cancer screening practices in primary care and behavioral health settings. Patients with CUD often present with overlapping social and behavioral risk factors, including tobacco use, alcohol consumption, and reduced engagement in preventive healthcare (Stinson et al., 2006). Given the potential compounding effects of these factors, routine oral examinations and risk stratification may be warranted in this population, even in the absence of overt symptoms. The observed relationship between CUD and oral cancer underscores the importance of integrating oral health awareness into substance use disorder treatment and counseling, especially as cannabis use becomes increasingly normalized.

Although CUD provides a clinically recognized marker of sustained and problematic cannabis use, it does not convey information on frequency, duration, or cumulative exposure. These parameters may be important in clarifying dose-response relationships and mechanisms underlying risk. In this cohort, use pattern data were not systematically recorded in structured fields and were therefore unavailable for analysis. Future investigations should incorporate detailed measures of cannabis exposure to improve risk estimation and interpretability.

From a public health perspective, this study adds to growing evidence that challenges the perception of cannabis as a risk-free substance. While therapeutic cannabinoids have demonstrated benefits in select contexts, such as chemotherapy-induced nausea or chronic pain management, the risks associated with habitual smoking of high-potency cannabis products remain underexplored (Volkow et al., 2016). As more states legalize recreational cannabis, longitudinal monitoring of health outcomes, including cancer incidence, will be essential to guide policy. Future studies should aim to disentangle the effects of cannabis mode of delivery (smoked vs. ingested), potency, and frequency of use on long-term cancer risk, and explore whether alternative delivery systems mitigate the risks associated with combustion.

## 5 Limitations

Limitations of this study include reliance on diagnostic codes, which may underrepresent both cannabis exposure and oral cancer incidence. In addition, information about methods of administration was not available for analysis, despite the potential utility of these data given recent increases in access to noncombustible forms of ingestion. Furthermore, we lacked data on important oral cancer risk factors, including alcohol consumption, human papillomavirus infection, and socioeconomic status. However, the use of a fixed follow-up window and uniform index date limits temporal bias, and our large sample size enhances statistical power and generalizability within clinical settings.

## 6 Conclusion

Our findings add to a growing body of literature suggesting that chronic or problematic cannabis use may contribute to cancer risk in tissues exposed to combustion products. As cannabis becomes more widely available and socially accepted, it is critical that public health messaging and clinical guidelines reflect emerging evidence on potential harms. Clinicians should consider oral cancer screening and counseling as part of routine care for individuals with CUD, particularly those with additional risk factors.

## Clinical trial registration

Not applicable.

## CRediT authorship contribution statement

**Raphael E. Cuomo:** Writing – review & editing, Writing – original draft, Validation, Software, Methodology, Investigation, Formal analysis, Data curation, Conceptualization.

## Patient consent statement

Not applicable.

## Ethics approval

This study is exempt from human subject protection under IRB protocol 1,604,619–1 of the University of California Health System.

## Permission to reproduce material from other sources

Not applicable.

## Funding

None.

## Declaration of competing interest

The authors declare that they have no known competing financial interests or personal relationships that could have appeared to influence the work reported in this paper.

## Data Availability

The data that support the findings of this study are derived from the University of California Health Data Warehouse (UCHDW), which consists of de-identified patient information. Due to the sensitive nature of the clinical data and the agreements under which they were obtained, the data cannot be made publicly available.
